# Supplementary material for: Reduced turnaround times through multi-sectoral community collaboration during the first surge of SARS-CoV-2 and associated effect on patient care and hospital operations
Source: PLoS One. 2021 Oct 7;16(10):e0257302. doi: 10.1371/journal.pone.0257302 (PMC8496830; doi:10.1371/journal.pone.0257302)
Supplement: S1 File — (DOCX) [file pone.0257302.s001.docx]

**Reduced turnaround times through multi-sectoral collaboration during the first surge of SARS-CoV-2 in Louisiana, March-April 2020: Supplemental Information**

**Supplemental Figure S1: Model Schematic:** Shows the flow of patients and the parameters governing such where admissions are moved into the COVID19 unit according to the turnaround time (TAT) and leave the COVID19 unit upon discharge (LoS) or a subsequent negative test.


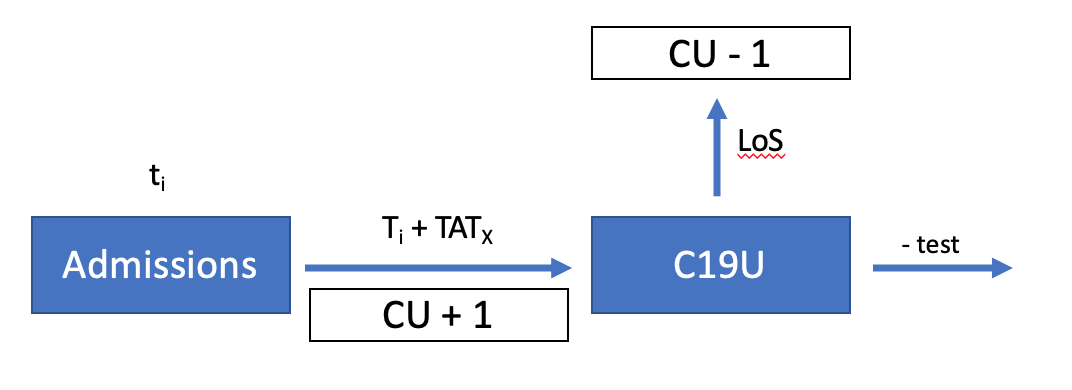


**Supplemental Figure S2:** Percent C19U positive individuals according to admitted for testing date.


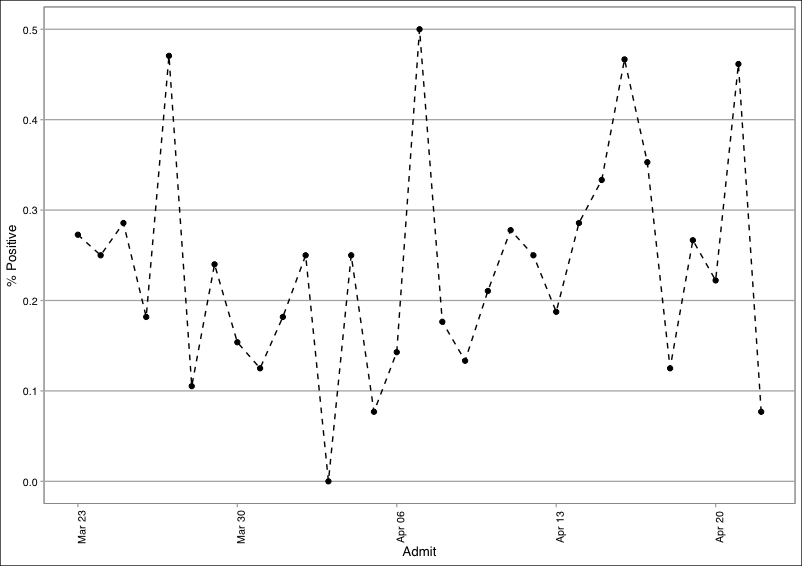


**Figure S3**: Turnaround time for non-RRTL tests during the RRTL offline period. There were two tests in each of the 9 and 13 day TAT, but these were considered outliers. Though shown here, only TAT between 4 and 7 days were used in the modeling effort.


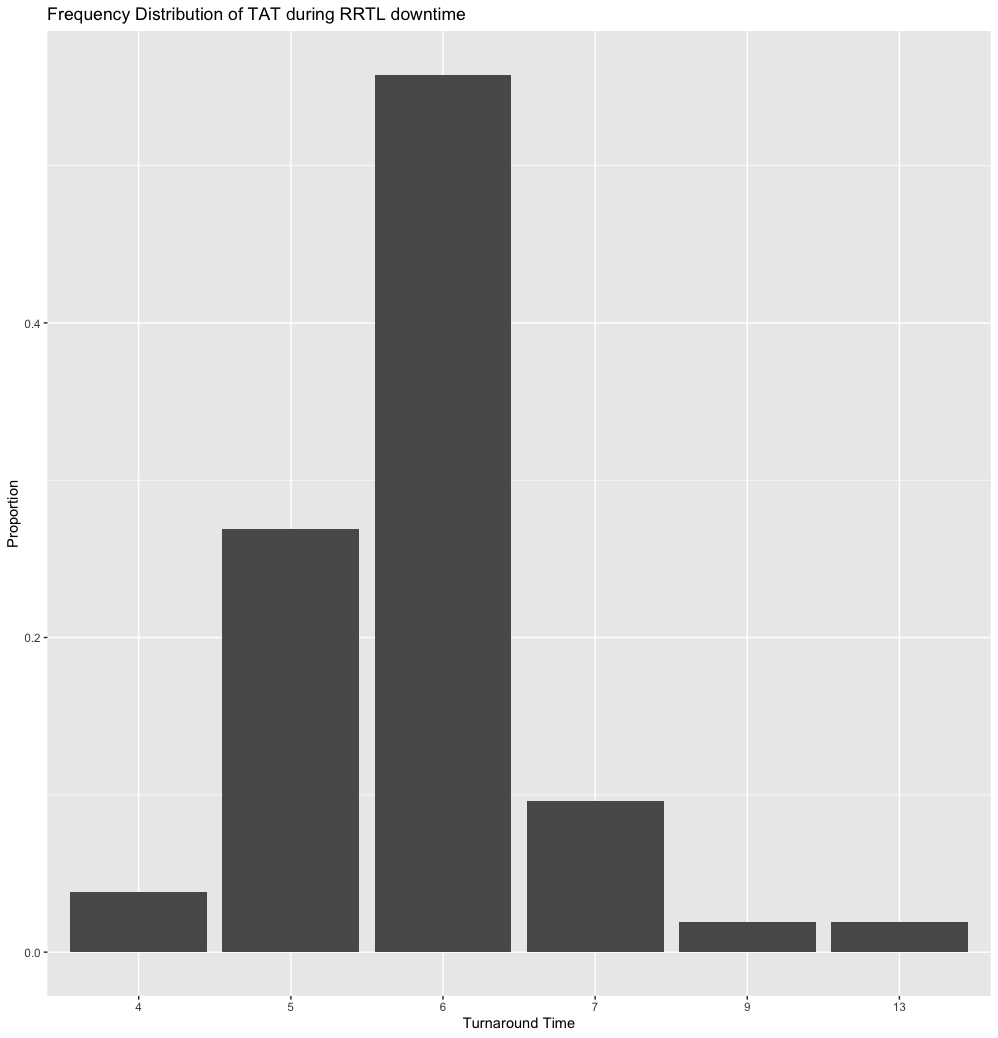


**Supplemental Figure S4:** A) Box plot showing the distribution of length of stay binned with the proportion of patients on the y-axis. B) Individual percentages of LoS from the patients in our data and C) Boxplot with the IQR of [2,10].


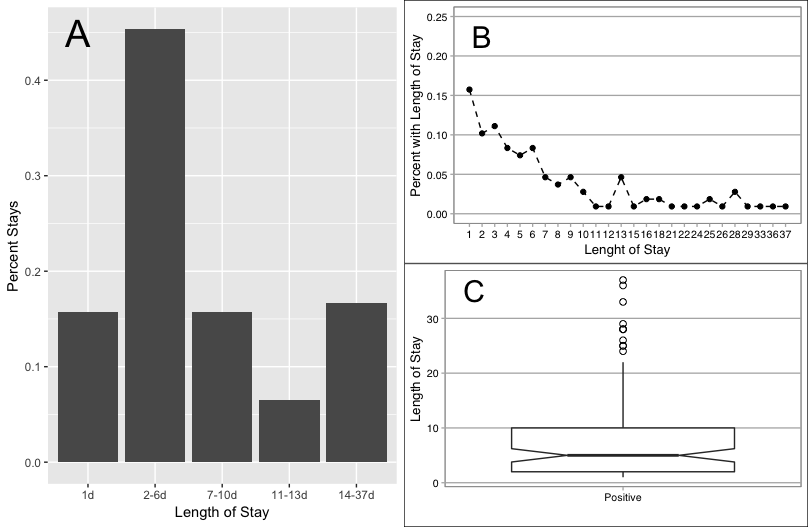


**Table S1**: Length of stays from 108 sampled inpatients determined by record examination and binned into categories based on similarity of proportions.

| Length of stay (days) | proportion | bin |
| --- | --- | --- |
| 1 | 0.16 | 1 |
| 2 | 0.10 | 2 |
| 3 | 0.11 | 2 |
| 4 | 0.08 | 2 |
| 5 | 0.07 | 2 |
| 6 | 0.08 | 2 |
| 7 | 0.05 | 3 |
| 8 | 0.04 | 3 |
| 9 | 0.05 | 3 |
| 10 | 0.03 | 3 |
| 11 | 0.01 | 4 |
| 12 | 0.01 | 4 |
| 13 | 0.05 | 4 |
| 15 | 0.01 | 5 |
| 16 | 0.02 | 5 |
| 18 | 0.02 | 5 |
| 21 | 0.01 | 5 |
| 22 | 0.01 | 5 |
| 24 | 0.01 | 5 |
| 25 | 0.02 | 5 |
| 26 | 0.01 | 5 |
| 28 | 0.03 | 5 |
| 29 | 0.01 | 5 |
| 33 | 0.01 | 5 |
| 36 | 0.01 | 5 |
| 37 | 0.01 | 5 |
